# Regulation of the JAK2-STAT5 Pathway by Signaling Molecules in the Mammary Gland

**DOI:** 10.3389/fcell.2020.604896

**Published:** 2020-11-17

**Authors:** Min Tian, Yingao Qi, Xiaoli Zhang, Zhihui Wu, Jiaming Chen, Fang Chen, Wutai Guan, Shihai Zhang

**Affiliations:** ^1^Guangdong Province Key Laboratory of Animal Nutrition Control, College of Animal Science, South China Agricultural University, Guangzhou, China; ^2^College of Animal Science and National Engineering Research Center for Breeding Swine Industry, South China Agricultural University, Guangzhou, China; ^3^Guangdong Laboratory for Lingnan Modern Agriculture, South China Agricultural University, Guangzhou, China

**Keywords:** Stat5, JAK2, signaling molecules, milk production, mammary gland development

## Abstract

Janus kinase 2 (JAK2) and signal transducers and activators of transcription 5 (STAT5) are involved in the proliferation, differentiation, and survival of mammary gland epithelial cells. Dysregulation of JAK2-STAT5 activity invariably leads to mammary gland developmental defects and/or diseases, including breast cancer. Proper functioning of the JAK2-STAT5 signaling pathway relies on crosstalk with other signaling pathways (synergistically or antagonistically), which leads to normal biological performance. This review highlights recent progress regarding the critical components of the JAK2-STAT5 pathway and its crosstalk with G-protein coupled receptor (GPCR) signaling, PI3K-Akt signaling, growth factors, inflammatory cytokines, hormone receptors, and cell adhesion.

## Introduction

The mammary gland is a critical organ in mammals and is involved in milk production and delivery. The mammary gland is a derivative of the skin, develops early during the embryonic stage and further develops and differentiates into a functional mammary gland during pubertal and adult stages (Gjorevski and Nelson, 2011). The development of the embryonic mammary gland starts with the formation of placodes, which then invaginate the mesenchyme and form mammary gland buds (Robinson, 2008). These buds continue to elongate and bifurcate, developing into a rudimentary gland prior to birth. Subsequently, the rudimentary mammary gland enters a quiescent phase and grows isomorphic with the body. The second stage of mammary gland development is initiated at puberty. During this period, mammary gland development is regulated and sustained by hormones, growth factors and cytokines. The tips of the rudimentary ducts transform into terminal end buds (TEBs) and penetrate into the mammary fat pad (Hinck and Silberstein, 2005). The mature mammary duct is mainly composed of myoepithelial cells (outer layer) and luminal epithelial cells (inner layer). During gestation, these mammary epithelial cells differentiate into milk-producing secretory alveoli, which synthesize the majority of milk fat, protein and lactose (Brisken et al., 1999; Oakes et al., 2008). The mammary gland rapidly undergoes involution after weaning, and approximately 80% of the epithelium is removed via apoptosis (Alexander et al., 2001; Watson, 2006).

Hormones are crucial for ductal morphogenesis in the mammary gland. During puberty, estrogen, growth hormone (GH) and prolactin are required for the development of the mammary gland (McNally and Martin, 2011). Estrogens are mainly secreted by the ovary and sensed by estrogen receptors (ERs), which are nuclear receptors. ERs regulate the transcription of multiple genes with a variety of coregulators [such as steroid receptor coactivator 1 (SRC-1) and Cbp/p300-interacting transactivator 1 (CITED1)] in the mammary gland (Howlin et al., 2006a,b). In contrast to estrogen, GH and prolactin regulate mammary gland function through the phosphorylation of Janus kinase 2 (JAK2) and activation of its downstream regulator signal transducers and activators of transcription 5 (STAT5) (Ihle, 1996). JAK2-STAT5 is proposed to be a critical signaling pathway in the mammary gland. In addition to the abovementioned hormones, JAK2-STAT5 is also regulated by cytokines such as IL-12, INF-γ, IL-4, IL-13, and IL-6. Recent studies have provided additional evidence that other prominent cellular signaling pathways (GPCR, PI3K/Akt and cell adhesion) might also be involved in crosstalk with JAK2-STAT5. The signaling pathways that interact with JAK2-STAT5 are overwhelmingly complex. In this review, we focus on the constitutive and extensive communication between JAK2-STAT5 and other signaling pathways.

## STATs and JAKs in the Mammary Gland

In the mammary gland, five STATs (STAT1, 3, 5a,b, and 6) have been identified (Watson and Neoh, 2008). STAT1 and STAT6 have been reported to play minor roles in the mammary gland. Although STAT1 is highly activated in the mammary gland, STAT1 knockout does not significantly affect ductal or alveolar morphogenesis (Klover et al., 2010). STAT6 is downstream of IL-4 and IL-3 and partially regulates the development of the alveoli. However, mice lacking STAT6 are still able to lactate (Khaled et al., 2007). In contrast to STAT1 and STAT6, STAT5, and STAT3 are the key STATs in the mammary gland. STAT5 promotes the proliferation of mammary gland epithelial cells, while STAT3 regulates the process of apoptosis during involution (Chapman et al., 2000). During late pregnancy and lactation, STAT5 is highly activated, as high levels of STAT5 can be detected in the nucleus in epithelial cells of the mammary gland, whereas STAT5 levels are undetectable during the involution period (Bednorz et al., 2011). STAT5 is involved in the side branching and maturation of alveolar cells (Vafaizadeh et al., 2010). Conditional inhibition of STAT5 in the mammary gland at different times further reveals its roles during specific periods of lactation (Reichenstein et al., 2011). Knocking out STAT5 during the first 3 days of lactation affects the expression of ER and connexin 32 (C × 32, a gap junction protein). STAT5 knockout during the first 10 days of lactation decreases neonatal body weight by 30–40% due to changes in mammary gland morphology and a reduction in milk production. Two isoforms of STAT5 (STAT5a and STAT5b) have been identified in the mammary gland (Liu et al., 1996). STAT5a and STAT5b are encoded by two separate genes located on chromosome 11 (mouse) and chromosome 17 (human). These genes are highly homologous (96% conserved at the protein level) and contain different C-terminal regions (Rani and Murphy, 2016). Knocking out STAT5a inhibits the normal development and differentiation of the mammary gland during pregnancy, whereas deletion of STAT5B only impairs body growth (Cui et al., 2004). After weaning, the phosphorylation of STAT5 is significantly decreased with increased phosphorylation of STAT3 (Watson and Neoh, 2008). The switch from the activation of STAT5 to STAT3 indicates the triggering of mammary gland involution. Activated STAT5 and STAT3 can enter the nucleus and regulate related gene expression. STAT5 is thought to regulate genes related to milk protein synthesis (α-casein) and other genes with unclear biological functions (kallikrein-8, prosaposin and Grb10) (Clarkson et al., 2006). In addition, activated STAT5 also regulates ACC1 expression by binding to its promoter and initiating *de novo* synthesis of fatty acids (Mao et al., 2002). Consistently, knocking down STAT5 decreases the expression of ACC1 (Li et al., 2019). It is still not clear whether STAT5 also regulates ACC2. This evidence indicates that STAT5 plays a vital role in milk synthesis. As expected, STAT3 regulates apoptosis by targeting the apoptosis regulator genes CCAAT enhancer binding protein-δ and c-Fos and regulating the PI3K/Akt signaling pathway (Clarkson et al., 2006).

Two isoforms of JAK (JAK1 and JAK2) are expressed in the mammary gland, and these factors are upstream of STAT. The biological functions of these two JAKs are somewhat different. Briefly, prolactin mainly regulates STAT5 activation through JAK2, while JAK1 is primarily regulates STAT3 activation (Xie et al., 2002). It is worth noting that JAK2 not only binds to the prolactin receptor but can also enter the nucleus. The potential mechanisms by which JAK2 regulates nuclear gene expression in the mammary gland are by modulating tyrosine kinase activity and preventing protein degradation. For example, JAK2 interacts with transcription factor nuclear factor 1-C2 (NF1-C) and enhances its stability, which further regulates the expression of genes involved in milk synthesis (Nilsson et al., 2006).

## Novel Factors in JAK2-STAT5 Activation

**FIGURE 1:**
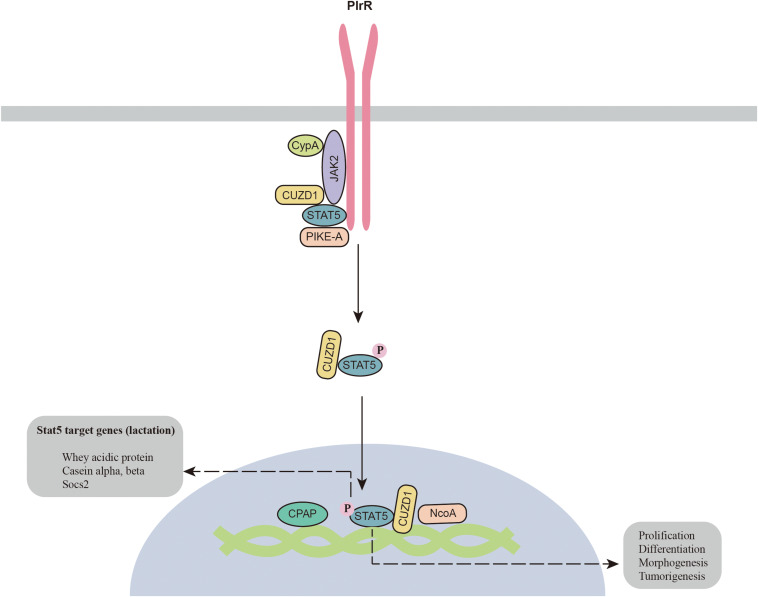
Novel components of the JAK2-STAT5 signaling pathway. CPAP, centrosomal P4.1-associated protein; CypA, cyclophilin A; CUZD1, zona pellucida-like domain-containing protein 1; JAK2, Janus kinase 2; NcoA, nuclear receptor co-activator; PIKE-A, PI 3-kinase enhancer A; PlrR, prolactin receptor; STAT5, signal transducers and activators of transcription 5.

The JAK2-STAT5 signaling pathway was identified long ago in the mammary gland. Recent studies have shown that additional components are required for the activation of JAK2-STAT5 (Figure 1). A prolyl isomerase called cyclophilin A (CypA) has been found to be an essential component for JAK2-STAT5 activation. CypA knockout disrupts mammary gland morphogenesis and differentiation by inhibiting the JAK2-STAT5 pathway (Volker et al., 2018). CUB and zona pellucida-like domain-containing protein 1 (CUZD1) is the other component involved in the regulation of mammary gland differentiation. CUZD1 knockout abolishes STAT5 phosphorylation and impairs mammary ductal branching and alveolar development. However, CUZD1 overexpression in mammary epithelial cells increases STAT5 phosphorylation (Mapes et al., 2017). Immunoprecipitation results show that CUZD1 forms a complex with JAK2 and STAT5, and CUZD1 knockout disrupts the connection between JAK2 and STAT5 (Mapes et al., 2017). In addition, PIKE-A has also been reported to participate in complex formation with PRLR and STAT5, which is required for activation of the PRLR and STAT5 signaling pathways (Chan et al., 2010). Knockout of PIKE-A in HC11 mammary gland epithelial cells attenuates cell proliferation by inhibiting STAT5 activation and cyclin D1 expression. The other critical protein for STAT5 activation is zinc finger homeobox 3 (ZFHX3), which is highly expressed during lactation (Zhao et al., 2016). ZFHX3 knockout results in underdevelopment of the mammary gland with decreased PRLR expression and STAT5 phosphorylation. The underlying mechanism by which ZFHX3 regulates the STAT5 signaling pathway is still unknown. The zinc finger transcription factor Miz1, which contains an N-terminal POZ domain and zinc finger motifs, has also been reported to maintain the normal function of the mammary gland. Knockout of Miz1 disrupts the activation of STAT5 signaling through by disrupting intracellular transport and localization of PRLR and ERBB4 (Sanz-Moreno et al., 2014).

In addition, a number of cofactors for p-STAT5 have also been identified in the mammary gland. For example, centrosomal P4.1-associated protein (CPAP) interacts with STAT5 and enhances its activity (Peng et al., 2002). NCoA-1 is another coactivator of STAT5a that regulates the synergistic effects of glucocorticoid receptor and STAT5a on beta-casein expression (Litterst et al., 2003). In summary, JAK2-STAT5 is a complicated signaling pathway, as a number of proteins are required for its activation in the mammary gland. More studies are required to clarify how these components function to regulate the JAK2-STAT5 signaling pathway.

## JAK2-STAT5 Crosstalk With the GPCR Signaling Pathway

G protein-coupled receptors (GPCRs) are crucial pharmaceutical targets that account for 33% of the targets of Food and Drug Administration (FAD)-approved drugs (Hauser et al., 2017). GPCRs are activated by small carboxylic acid metabolites (GPR41, GPR43, GPR81, GPR109A, GPR109B, and GPR84), triglyceride metabolites (GPR40, GPR120, and GPR119), bile acids (GPBAR1), and amino acid and amino acid metabolites (GPR142, CasR, GPR35, TAAR1, and FBR1/2) (Husted et al., 2017). Dietary nutrients and their metabolites can regulate the development and lactation of the mammary gland through the activation of GPCR signaling pathways. In addition, a number of hormones (glucagon, luteinizing hormone, and epinephrine) and neurotransmitters (acetylcholine, dopamine, and serotonin) can also trigger the activation of corresponding GPCRs (Neves et al., 2002). Previously, glucagon, epinephrine and dopamine have been reported to be involved in the regulation of breast cancer (Wang et al., 2002; Ligumsky et al., 2012; Cui et al., 2019), while serotonin controls the development of the mammary gland (Matsuda et al., 2004). These receptors control multiple signaling cascades and regulate various physiological functions. Therefore, understanding the interplay between GPCRs and JAK2-STAT5 is crucial.

GPCRs are 7-transmembrane proteins that are coupled to heterotrimeric G proteins on the intracellular side of the membrane (Thal et al., 2018). The G protein contains G_α_ (binds to GTP/GDP), G_β_ and G_γ_ subunits (Wettschureck and Offermanns, 2005). The unique downstream signal activation of different GPCRs mainly relies on the classification of the G_α_ subunits. To date, four G_α_ subunits (G_αs_, G_αi_, G_αq/11_, and G_α 12/13_) have been identified in cells (Syrovatkina et al., 2016). G_αi_ and G_αs_ mainly participate in the regulation of cellular adenosine 3′,5′-cyclic monophosphate (cAMP) through adenylyl cyclase (AC) (Neves et al., 2002). cAMP is a critical second messenger that mainly regulates cellular biological functions through protein kinase A (PKA) and exchange proteins directly activated by PKA and cAMP (EPAC). G_αq_ can increase cellular Ca^2+^ signaling and activate protein kinase C (PKC)-dependent signaling pathways (Neves et al., 2002; Winzell and Ahrén, 2007).

**FIGURE 2:**
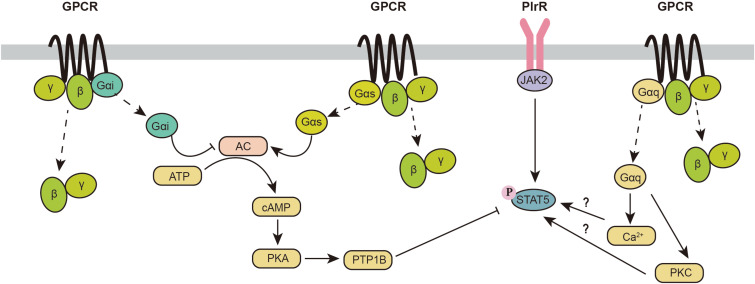
Crosstalk between the GPCR and JAK2-STAT5 signaling pathways. AC, adenylyl cyclase; cAMP, cellular adenosine 3’,5’-cyclic monophosphate; GPCR, G protein-coupled receptor; JAK2, Janus kinase 2; PKA, protein kinase A; PKC, protein kinase C; PlrR, prolactin receptor; PTP1B, protein-tyrosine phosphatase 1B; STAT5, signal transducers and activators of transcription 5.

Originally, it was reported that GPCR activation has minor effects on the JAK2-STAT5 signaling pathway. However, recent evidence indicates an intimate relationship between GPCR and JAK2-STAT5 signaling pathways (Figure 2). Activation of GPCRs coupled to the G_αq_ subunit has been reported to increase the phosphorylation of STAT5. The oxytocin receptor (OXTR) is a G protein-coupled receptor that binds to G_αq_. Overexpression of OXTR in the mouse mammary gland increases phosphorylation of STAT5 and induces lactation during the early lactation period, whereas OXTR-induced phosphorylation of STAT5 is decreased with attenuated milk production during the peak lactation period (Li et al., 2018). Furthermore, knockout of OXTR can impair milk ejection (Li et al., 2018). This finding indicates that the effect of the G_αq_ signaling pathway in the mammary gland may be dependent on different lactation periods. GPR54 is another GPCR coupled to G_αq_, which can be activated by kisspeptins (Kps). GPR54 is highly expressed during the lactation period. Activation of GPR54 increases β-casein synthesis in the mammary gland with activation of the mTOR, ERK1/2, and STAT5 signaling pathways (Kobayashi et al., 2016). In summary, these data provide some primary evidence for a potential link between the G_αq_ signaling pathway and JAK2-STAT5. However, the underlying mechanism involved in this process is not clear.

In addition to G_αq_, G_αs_- and G_αi_-related signaling pathways also participate in the phosphorylation of STAT5. As a direct downstream target of the G_αs_/_αi_ signaling pathway, increased cellular cAMP significantly decreases STAT5 phosphorylation and β-casein synthesis through an increase in protein-tyrosine phosphatase 1B (PTP1B) (Chiba et al., 2016). In addition, it has been reported that PKA inhibition inhibits the secretion of newly synthesized caseins (Clegg et al., 1998). PKA inhibits the vesicular structure of the Golgi body and inhibits casein production mainly through exocytosis (Clegg et al., 1998). Furthermore, PKA is also known to decrease protein synthesis through the inhibition of the mTORC1 signaling pathway (phosphorylation of raptor on Ser792) (Jewell et al., 2019). Because G_αs_ is a positive regulator of cAMP and G_αi_ is a negative regulator of cAMP, activation of the G_αs_ signaling pathway inhibits the stat5 signaling pathway, while triggering G_αi_ signaling can activate the STAT5 signaling pathway.

It is worth noting that many GPCRs are not coupled to a unique G_α_ protein, which makes the situation more complicated. For example, melatonin regulates mammary gland function through the melatonin receptors MT1 and MT2. MT2 is only coupled to a G_αi_ subunit, while MT1 is coupled to both G_αi_ and G_αq_ subunits (Tosini et al., 2014). Although G_αi_ and G_αq_ are thought to individually activate STAT5 in the mammary gland, overexpression of MT1 in the mammary gland surprisingly inhibits mammary gland development and milk synthesis, which is consistent with the decrease in STAT5 phosphorylation and the expression of estrogen and progesterone receptors (Xiang et al., 2012). One possible reason for this contradictory finding might be that the activation of GPCRs activates not only the G_α_ signaling pathway but also G_βγ_ subunits, regulating many downstream effector targets. At present, the effects of G_βγ_ on the phosphorylation of STAT5 in the mammary gland are still unclear. In more complicated situations, some GPCRs might be coupled to G_αs_ and G_αq_. Recent studies indicate that the activation of GPCRs could be biased (Qiao et al., 2020; Suomivuori et al., 2020). Thus, it is unrealistic to hypothesize which G_α_ subunits will be dominantly activated. More studies are needed to identify the effects of different GPCRs on the phosphorylation of STAT5.

## JAK2-STAT5 Crosstalk With the PI3K-Akt Signaling Pathway

During puberty, branching morphogenesis is initiated by GH, estrogen, and IGF1. Intriguingly, GH, estrogen and IGF-1 are all involved in the activation of the PI3K/Akt signaling pathway. Breast cancer is the most common health risk for women. Approximately two-thirds of breast cancers are hormone-dependent (Subramani et al., 2017). Estrogen and GH dysregulation are also closely related to breast cancer. Specifically, genetic ablation of p110α (a catalytic subunit of PI3K) inhibits tumor formation, while knocking out p110β enhances ductal branching and tumorigenesis (Utermark et al., 2012). This evidence suggests that PI3K/Akt signaling is involved in the regulation of normal mammary gland growth and breast cancer development.

Akt is an important regulator of mammary gland development and milk synthesis. During the gestation and lactation periods, total and phosphorylated Akt are significantly increased in the mammary gland and are significantly decreased during the involution period (Schwertfeger et al., 2001; Boxer et al., 2006). Three isoforms of Akt have been identified in the mammary gland. Different Akt subtypes seem to execute different functions. Knockout of Akt1 but not Akt2 or Akt3 interferes with the activation of STAT5, delays differentiation and promotes apoptosis in the mammary gland (Maroulakou et al., 2008). However, Chen et al. (2010) found that knocking out either Akt1 or Akt2 in mice still resulted in normal mammary epithelial differentiation and STAT5 activation. Knockout of one allele of Akt2 in Akt1-deficient mice significantly blocks the phosphorylation of STAT5, which leads to defects in mammary gland differentiation and milk production. This evidence indicates that Akt isoforms might play overlapping regulatory roles and are critical in the mammary gland. Importantly, activation of the PI3K-Akt pathway triggers autocrine-mediated prolactin secretion, which indirectly activates the JAK2-STAT5 signaling pathway. This process is required for the initiation of lactation (Oliver and Watson, 2013). Future studies are needed to verify whether Akt1 and Akt2 are both required for the activation of the JAK2-STAT5 signaling pathway.

**FIGURE 3:**
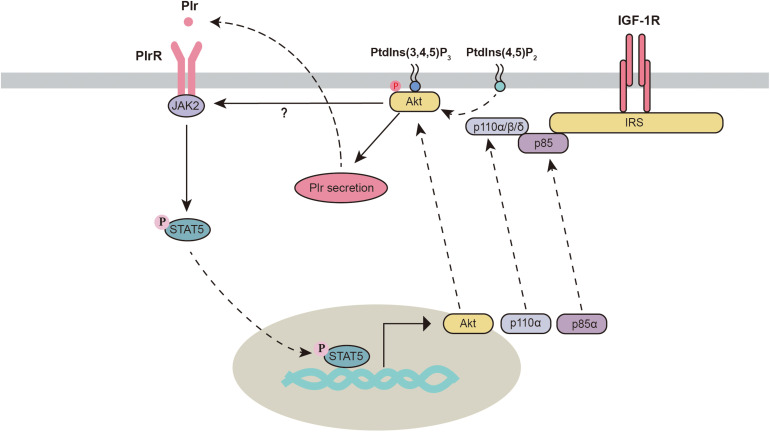
Crosstalk between the PI3K/Akt and JAK2-STAT5 signaling pathways. Akt, protein kinase B; IGF-1R, insulin-like growth factor 1 receptor; IRS, insulin receptor substrate; JAK2, Janus kinase 2; Plr, prolactin; PlrR, prolactin receptor; Ptdlns(3,4,5)P_3_, phosphatidylinositol 3,4,5-trisphosphate; Ptdlns(4,5)P_2_, phosphatidylinositol-4,5-bisphosphate; STAT5, signal transducers and activators of transcription 5.

In the mammary gland, JAK2 deficiency decreases mammary gland cell proliferation, which can be partially recused by the overexpression of Akt1 (Sakamoto et al., 2007), suggesting a potential link between JAK2 and Akt1. In addition, activation of STAT5 can also directly regulate PI3K-Akt1 signaling through the mechanism described below (Figure 3). First, Stat5 directly binds to consensus sites within Akt1 and enhances its transcriptional activation (Creamer et al., 2010; Schmidt et al., 2014). Second, stat5 increases the transcription of two subunits (p85α and p110α) of phosphatidylinositol 3-kinase (PI3K) in the mammary gland (Schmidt et al., 2014). Third, STATs can regulate the activity of PI3K by binding to the p85 regulatory subunit (Rosa Santos et al., 2000; Nyga et al., 2005). This evidence indicates strong crosstalk between the PI3K-Akt and JAK2-STAT5 signaling pathways.

## JAK2-STAT5 Crosstalk With Growth Factors and Inflammatory Cytokines

**FIGURE 4:**
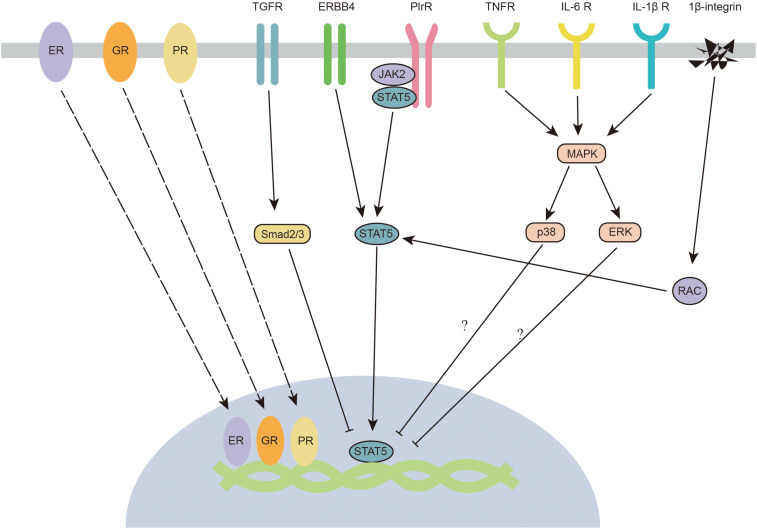
Crosstalk between JAK2-STAT5 and hormone receptors, growth factors, inflammatory cytokines, and integrins. ER, estrogen receptor; ERBB4, epidermal growth factor receptor 4; ERK, extracellular signal-regulated kinase; GR, glucocorticoid receptor; IL-1β R, interleukin-1beta receptor; IL-6 R, interleukin-6 receptor; JAK2, janus kinase 2; MAPK, mitogen-activated protein kinases; PlrR, prolactin receptor; PR, progestinreceptor; Smad2/3, STAT5, signal transducers and activators of transcription 5; TGFR, transforming growth factor receptor; TNFR, tumour necrosis factor receptor.

Growth factors are crucial elements involved in JAK2/STAT5 regulation in the mammary gland (Figure 4). Transforming growth factor-β (TGF-β) is considered a crucial factor in the regulation of mammary gland development, as well as mammary tumorigenesis. TGF-β regulates mammary gland epithelial cells through an autocrine mechanism. Three isoforms of TGF-β (TGF-β1, TGF-β2, and TGF-β3) have been identified in mammals. All isoforms negatively regulate the development of the mammary gland (Daniel and Robinson, 1992). TGF-β3 is significantly increased during the involution process in the mouse mammary gland (Faure et al., 2000). A high concentration of TGF-β inhibits the branching process of the mammary gland (Nelson et al., 2006), whereas ductal proliferation and lateral branching are highly increased in TGF-β-mutant mice (Joseph et al., 1999; Crowley et al., 2005). The imbalance between non-canonical and canonical TGF-β signaling leads to mammary tumorigenesis (Parvani et al., 2011). The canonical downstream targets of TGF-β signaling are Smads, which are a group of transcription factors. TGFβ-induced Smad signaling (smad2/3/4 complex) antagonizes prolactin-mediated JAK/STAT signaling by blocking STAT5 transactivation of its target genes (Cocolakis et al., 2008). Intriguingly, TGFβ is usually highly expressed during the middle of lactation. The antagonistic effect of TGFβ is partially reduced by SnoN, which is an inhibitor of smad proteins (Jahchan et al., 2012). In addition, TGF-β might also regulate the function of the mammary gland via the activation of the non-canonical WNT5A pathway (Roarty and Serra, 2007).

Epidermal growth factor (EGF) is an important factor for mammary gland development (Long et al., 2003). EGF receptors (ERBBs) belong to the tyrosine kinase family, and four ERBBs have been identified (ERBB1, ERBB2, ERBB3, and ERBB4) in the mammary gland. ERBB1 and ERBB4 have been reported to participate in the regulation of mammary gland development. When ERBB1 is knocked out, alveolar development is severely impaired (Fowler et al., 1995). Intriguingly, it has been proposed that ERBB1 is mainly located in the stroma but not in the epithelium (Wiesen et al., 1999; Gallego et al., 2001). These findings suggest an intimate reciprocal stromal-epithelial interaction in the mammary gland. In contrast to ERBB1, the absence of ERBB4 leads to a deficiency in milk secretion (Tidcombe et al., 2003). Specifically, knocking out ERBB4 in mammary epithelial cells significantly impairs the differentiation and proliferation of cells in the mammary gland (Long et al., 2003). ERBB4 tyrosine kinases might also act as scaffold proteins that interact with JAK2 and STAT5 (Muraoka-Cook et al., 2008). When activated, ERBB4 is cleaved at Val-675 and releases a soluble 80-kDa intracellular domain (s80HER4). The kinase activity of s80HER4 is also required for the nuclear translocation of STAT5A (Muraoka-Cook et al., 2006). Thus, similar to PRLR, stimulation of ERBB4 triggers the activation of STAT5. Although some studies indicate cooperative crosstalk between prolactin and EGF (Darcy et al., 1995), other studies showed an antagonistic relationship between these factors (Fenton and Sheffield, 1997; Huang et al., 2006). EGF might inhibit PRL-induced mammary gland functions by modifying STAT5-mediated gene expression. In addition, EGF blocks the STAT5-induced pathway through growth factor receptor-bound protein 2 (Grb2), which is a positive regulator of cell proliferation during morphogenesis (Brummer et al., 2006).

In the context of mammary gland infection by pathogens during lactation, multiple inflammatory cytokines (TNF-α, IL-1β, and IL-6) are released by immune cells in the mammary gland and impair milk production (Shuster et al., 1996; Quesnell et al., 2012). IL-1β and TNF-α have been reported to inhibit milk protein production (β-casein expression) through activation of the NF-κB signaling pathway (Bonizzi and Karin, 2004). NF-κB and AMPK are two critical downstream signals of inflammatory cytokines (TNF-α, IL-1β, and IL-6). To date, JAK2 has been demonstrated to induce the phosphorylation of the inhibitor of NF-κB (Digicaylioglu and Lipton, 2001). However, no evidence indicates that NF-κB can regulate the activation of AMPK, which indicates that this field still needs further research. Interestingly, some experimental clues in non-mammary gland epithelial cells suggest that P38/ERK MAPK signaling pathways might be involved in the regulation of STAT5 (Figure 4). ERK has been reported to inhibit the transcriptional activity of STAT (Krasilnikov et al., 2003). Furthermore, the phosphorylation of p38 is negatively correlated with the phosphorylation of STAT5 (Gaoxia et al., 2018). The potential effects of P38/ERK MAPK of STAT5 in mammary gland epithelial cells still require further study. Recently, TNF-α has been shown to significantly inhibit lactose synthesis by inactivating JAK2 in the mammary gland (Kobayashi et al., 2016). It would be interesting to know if other inflammatory cytokines also affect the activation of the JAK2-STAT5 signaling pathway.

## JAK2-STAT5 Crosstalk With Hormone Receptors

As we previously mentioned in the “JAK2-STAT5 and PI3K-Akt” section, numerous hormones indirectly regulate JAK2-STAT5 through the PI3K/Akt signaling pathway. In this section, we summarize certain hormone receptors that can directly regulate the JAK2-STAT5 signaling pathway. Prolactin is one of the most predominant hormones that regulates mammary gland development (Saunier et al., 2003). The prolactin receptor-dependent signaling pathway is critical for the proliferation and differentiation of mammary alveoli during gestation (Miyoshi et al., 2001). It is widely known that prolactin is mainly responsible for the activation of JAK2-STAT5. In addition to the prolactin receptor, glucocorticoid, estrogen, and progestin receptors have also been reported to directly regulate the JAK2-STAT5 signaling pathway (Figure 4). In the mammary gland, glucocorticoid administration can increase milk protein synthesis through glucocorticoid receptors. The glucocorticoid receptor has been reported to interact with STAT5a and enhance STAT5a-mediated gene transcription. Both glucocorticoid receptor and STAT5a recruit the histone acetyltransferase (HAT) p300 coactivator (Pfitzner et al., 1998; Kabotyanski et al., 2006). Intriguingly, a recent study indicated that glucocorticoid receptors regulate beta-casein gene expression by directly interacting with a proximal promoter and a distal enhancer, forming a chromatin loop that connects the promoter and enhancer (Kabotyanski et al., 2009). This chromatin loop is important in regulating milk synthesis gene expression. Similar to glucocorticoid receptors, estrogen and progestin receptors have also been proposed to regulate STAT5 by interacting with the DNA domain. Estrogen receptor-α and -β enhance prolactin-induced STAT5 activation by directly binding to the STAT5 DNA-binding domain (Björnström et al., 2001; Faulds et al., 2001). The crosstalk between the progestin receptor and the PRLR/STAT5 signaling pathway occurs at the β-casein promoter. Progestin-induced activation of progestin receptor leads to direct binding of progestin receptor to the beta-casein promoter and blocks its activation, which might lead to an inactivated form of STAT5a (Richer et al., 1998).

Recently, some preliminary evidence has indicated that hormones such as insulin, serotonin and leptin also participate in the regulation of the JAK2-STAT5 signaling pathway. Insulin plays an important role in enhancing milk synthesis by phosphorylating STAT5 in the mammary gland (Menzies et al., 2010). The insulin receptor can directly interact with STAT5 and induce its phosphorylation (Chen et al., 1997). In addition, one of the critical downstream signaling pathways of insulin is thought to increase the activity of the PI3K/KAT signaling pathway, which is a critical signaling pathway that crosstalks with JAK2-STAT5. Serotonin inhibits the phosphorylation of STAT5 and decreases β-casein expression (Chiba et al., 2014). Although leptin has not been reported to directly crosstalk with the STAT5 signaling pathway in the mammary gland, it is thought to synergize with prolactin to enhance the expression of beta-casein in the mammary gland through the inactivation of STAT3 (Motta et al., 2007).

## Integrins and JAK2-STAT5

Cell adhesion is a critical factor that determines the fate of epithelial cells (Schmidt et al., 1993). As major receptors associated with cell adhesion, integrins have been reported to regulate cell proliferation, differentiation and migration (Díaz-Coránguez et al., 2019). β1-integrin is thought to maintain the function of the mammary gland via the integrin-containing adhesion complex protein ILK (integrin-linked kinase). ILK regulates STAT5 signaling through Rac1 (Akhtar et al., 2009), which (RAS-related C3 botulinum substrate 1) is a critical downstream factor of integrins (Akhtar and Streuli, 2006; Figure 4). Mechanistically, Rac1 recruits STAT5 to kinase complexes and enhances its phosphorylation (Xu et al., 2010). Knocking out β1-integrin decreases the activation of STAT5, impairs the differentiation of secretory epithelial cells, and inhibits the mRNA expression of beta-casein and whey acidic protein (Faraldo et al., 2002).

## Other Signaling Pathways and the Activation of JAK2-STAT5

In addition to the abovementioned pathways that interact with JAK2-STAT5, other signaling pathways are involved in the regulation of JAK2-STAT5. (1) The Hedgehog signaling pathway negatively regulates mammary gland development. Overexpressing the Hedgehog effector protein GLI1 attenuates the expression of STAT5 through snail and inhibits mammary gland lactation (Fiaschi et al., 2007). (2) Peroxisome proliferator-activated receptor γ (PPARγ) has been shown to regulate STAT5A protein expression (Olsen and Haldosen, 2006). (3) NF-kappa B functions as a negative regulator of the JAK2-STAT5 pathway by interfering with STAT5 tyrosine phosphorylation (Geymayer and Doppler, 2000). However, more evidence is required to support the interplay between JAK2-STAT5 and these signaling pathways.

## Conclusion and Perspective

Signal transducers and activators of transcription 5 is a crucial transcription factor that directly regulates multiple genes that participate in proliferation, differentiation, and milk secretion in the mammary gland. The current understanding of the crosstalk between JAK2-STAT5 and other signals includes the following: (1) activation of the G_αi_ or G_αq_ GPCR signaling pathway is thought to increase the phosphorylation of STAT5, and the effects of different types of GPCRs could be different due to the bias of agonists; (2) Akt1 activates STAT5 phosphorylation, which can increase the expression of Akt1 and PI3K subunits (p85α and p110α); (3) TGF-β, TNF-α, IL-6, and IL-1β are negative regulators of STAT5 activation, while the effects of EGF on the mammary gland are still controversial; (4) ER, GR and PR are positive regulators of the JAK2-STAT5 signaling pathway by directly interacting with the DNA domain; and (5) cell adhesion is crucial in maintaining the PrlR/STAT5 signaling cascade through β1-integrin.

It is worth noting that in addition to its function in mammary gland epithelial cells, the STAT5 signaling pathway also plays an important role in macrophages in the mammary gland and is required for normal mammary gland development. STAT5 knockout in macrophages leads to decreased ductal elongation but increased epithelial cell proliferation. Mechanistically, STAT5 deletion induces the expression of the proliferative factors Cyp19a1/aromatase and IL-6, which enhance ER signaling in the mammary gland (Brady et al., 2017). It would be interesting to know whether the STAT5 signaling pathway also plays a crucial role in other cell types (fibroblasts, adipocytes, blood vessels, nerves, and various immune cells) in the mammary gland. With the development of single-cell RNA sequencing, it would be possible to identify the potential signals that crosstalk with JAK-STAT5 in individual cells in the mammary gland. More research on the crosstalk among different types of cells in the mammary gland would help us to better understand the signaling networks in the whole mammary gland.

Mammary gland development and lactation are complicated processes that are accompanied by magnificent changes in reproductive hormones. During lactation, mastitis occurs widely and causes inflammatory injury of the mammary gland. Although various signaling interactions have been identified between JAK2-STAT5 and reproductive hormones, growth factors and inflammatory cytokines, the potential challenge in the future is to precisely predict the biological modifications in the mammary gland mediated by these combinatorial signaling activities.

## Author Contributions

MT and SZ initiated the idea, the scope, and the outline of this review manuscript. MT, SZ, YQ, XZ, ZW, JC, and FC studied and analyzed all of the publications cited in this manuscript and were involved in the manuscript preparation. SZ and WG conducted the final editing and proofreading. All authors read and approved the final manuscript.

## Conflict of Interest

The authors declare that the research was conducted in the absence of any commercial or financial relationships that could be construed as a potential conflict of interest.
